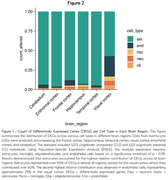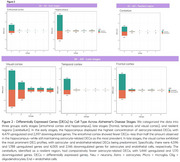# Cellular signatures of vulnerability and resilience in Alzheimer's Disease brains

**DOI:** 10.1002/alz70855_096282

**Published:** 2025-12-23

**Authors:** Gabriela Mantovani Baldasso, Christian Limberger, Sofia Gallo Salvadori, Rodrigo Sebben Paes, Marco Antônio De Bastiani, Eduardo R. Zimmer

**Affiliations:** ^1^ Universidade Federal do Rio Grande do Sul, Porto Alegre, Rio Grande do Sul, Brazil; ^2^ Brain Institute of Rio Grande do Sul, Porto Alegre, Brazil; ^3^ McGill Centre for Studies in Aging, Montreal, QC, Canada

## Abstract

**Background:**

Neurodegeneration is a critical pathological feature of Alzheimer's disease (AD). This process not only affects neurons but also involves glial cells. However, the biological mechanisms underlying the cellular vulnerability and resilience to neurodegeneration in AD remain unclear. Therefore, we aimed to investigate the transcriptomic brain cell type signatures in vulnerable or resilient brain regions of healthy and AD brains.

**Method:**

We searched microarray AD datasets in the Gene Expression Omnibus repository for 21 available transcriptomic data from postmortem tissue, including the following regions: frontal cortex, hippocampus, temporal cortex, visual cortex, entorhinal cortex, and cerebellum. The datasets included 1,073 cognitively unimpaired (CU) and 1,521 cognitively impaired (CI) individuals. We performed gene expression deconvolution with the Population‐Specific Expression Analysis (PSEA) package to obtain the differentially expressed genes (DEGs) specific for neurons, astrocytes, microglia, oligodendrocytes, and endothelial cells (FDR‐adjusted *p*‐value < 0.05). All analyses were done in R.

**Result:**

The PSEA analysis revealed a higher percentage of DEGs in astrocytes, followed by endothelial cells compared to neurons, microglia and oligodendrocytes in all regions (Figure 1). The hippocampus showed a higher number of astrocyte‐related DEGs in brain regions associated with early stages of AD (Figure 2A), while in late stages a higher number of DEGs was found in the visual cortex (Figure 2B). Although the cerebellum had some astrocyte‐related DEGs, its percentages were lower (Figure 2C).

**Conclusion:**

Our findings reveal that astrocytes are the cell type consistently exhibiting a higher number of DEGs across all brain regions. Other cells show a similar percentage of DEGs in all investigated brain areas – both vulnerable and resilience regions. These results indicate that astrocytes may develop distinct phenotypes across brain regions during AD progression, which may be associated with the degree of amyloid‐beta and tau accumulation in the brain.